# Effect of Natural Turf, Artificial Turf, and Sand Surfaces on Sprint Performance. A Systematic Review and Meta-Analysis

**DOI:** 10.3390/ijerph17249478

**Published:** 2020-12-17

**Authors:** Javier Sanchez-Sanchez, Alejandro Martinez-Rodriguez, Jose Luis Felipe, Antonio Hernandez-Martin, Esther Ubago-Guisado, Jens Bangsbo, Leonor Gallardo, Jorge Garcia-Unanue

**Affiliations:** 1 School of Sport Sciences, Universidad Europea de Madrid, 28670 Madrid, Spain; javier.sanchez2@universidadeuropea.es; 2 Analytical Chemistry, Nutrition and Food Science Department, University of Alicante, 03690 Alicante, Spain; amartinezrodriguez@ua.es; 3 IGOID Research Group, Physical Activity and Sport Sciences Department, University of Castilla-La Mancha, 45071 Toledo, Spain; Antonio.HMartinSan@uclm.es (A.H.-M.); esther.ubago@gmail.com (E.U.-G.); Leonor.Gallardo@uclm.es (L.G.); Jorge.GarciaUnanue@uclm.es (J.G.-U.); 4 Department of Nutrition, Exercise and Sports, University of Copenhaguen, 2177 Copenhagen, Denmark; jbangsbo@nexs.ku.dk

**Keywords:** speed, fatigue, artificial turf, natural turf, sand, performance analysis

## Abstract

The aim of this study was to analyze the influence of natural turf, artificial turf, and sand on sprint performance in different sports and to determine how the sport surface affects sprint performance. A systematic search was conducted in Pubmed, Web of Sciences, and SPORTDiscus databases. Out of 5644 studies, 11 studies were included in the meta-analysis. The studies were very heterogeneous, as they examined different structural characteristics or quality parameters. The studies on natural turf and sand showed significant improvements on sprint speed during training. On the other hand, the analysis of fatigue did not reveal significant differences in the deterioration of sprint speed on both natural and artificial turf. Significance was set at *p* < 0.05. In conclusion, although lower performance in sprint was reported on sand, further studies are needed to explain the differences in sprint on natural and artificial turf.

## 1. Introduction

The main function of a sport surface is to ensure safety and adequate player performance during physical exercise practice [1]. One of the most important goals in sport surface construction is to improve sport performance [2]. The constant improvement of sport surfaces like artificial turf is motivated by the demands of the sport sector, as the quality of sport surfaces is considered a determining factor for achieving results [3]. It has been suggested that changes in sport surfaces can have effects on performance patterns and athlete injury [4]. Various studies have proven a relation between the elasticity of a sport surface and athletes’ performance [5].

Research on injury risk has shown contradictory results, although in general, the risk of injury appears to be equivalent on artificial turf and natural grass [6,7]. On the other hand, Plaza-Carmona et al. [8] have shown that bone mass is not affected by practicing football on natural or on artificial turf. Other studies have focused on biomechanical aspects. Alcaraz et al. [9] analyzed running speed kinetics on sand and on an athletic track, finding significant differences in players’ biomechanics. Previous researches have provided information on the influence of the structural components of artificial turf on its mechanical properties, like the study by Sánchez-Sánchez et al. [10], in which it was observed that a compact gravel sub-base extended surface durability, with adequate security parameters.

Likewise, exercise on soft surfaces, like sand, is associated with higher energy expenditure and lower stimulus in impact training than on hard surfaces [11,12,13]. Brito et al. [14] measured higher levels of lactate and increased heart rate in football players during a simulated match on sand compared to artificial turf and hard surfaces. Other studies recommend sand as a training surface for improving neuromuscular adaptations [12,15]. In volleyball players, vertical jump was higher on a hard surface than on a soft surface [16,17].

Another common sport activity analyzed on different sport surfaces is sprint. It has been proven that the high absorption that occurs on sand surfaces limits the maximum speed [11,18]. Therefore, physical performance in sprint and jumping actions is influenced by traction, rigidity, and force reduction of the surface [19]. Studies like the one by Brechue et al. [20] analyzed the differences in speed during sprints on sand and on the track, showing a significant speed reduction on sand. However, no significant differences have been demonstrated in speed on artificial and natural turf, except when the sprinting action includes changes in direction, in which case, speed is higher on artificial turf [21]. Despite this, controversy exists, as the physiological demands of sprint on artificial and natural turf were found to be similar in some studies [22] or higher on natural surfaces in other works [23].

The aim of this study was to analyze sprint performance on natural grass surfaces, artificial grass, and sand in football, rugby, hockey, and netball, through cross-correlation studies. A systematic literature review was performed, gathering athletes’ speed performance and fatigue protocols, to evaluate the influence of the surface.

## 2. Materials and Methods

### 2.1. Experimental Approach to the Problem

This study was completed in accordance to the Preferred Reporting Items for Systematic reviews and Meta-Analyses (PRISMA) guidelines [24]. A search strategy was developed to identify all relevant studies assessing the effect of different surfaces on sprint speed in athletes. The search was registered in PROSPERO. Our systematic search was conducted in different online databases: PubMed (whole database), SPORTDiscus, and Web of Science (whole database), since their inceptions until 17 March 2020. The terms used in the search of the databases were: (‘soccer’ OR ‘football’ OR ‘rugby’ OR ‘hockey’ OR ‘netball’) AND (performance OR assessment OR sprint OR speed) AND (‘artificial turf’ OR ‘synthetic’ OR ‘natural’ OR ‘grass’ OR ‘sand’ OR ‘playing surface’ OR field).

### 2.2. Study Selection

Studies included in our analysis were original research articles and had to meet the following criteria: (1) performed on athletes; (2) focused on the influence of the sport surface on sport performance; (3) used a test guaranteeing at least one completion of a maximum-intensity sprint; (4) compared the sprint speed (distance and time) of athletes on natural turf, artificial turf, and sand; (5) could include training methods; (6) incorporated at least two of the previous mentioned surfaces; (7) were published papers); (8) published in English.

**Figure 1:**
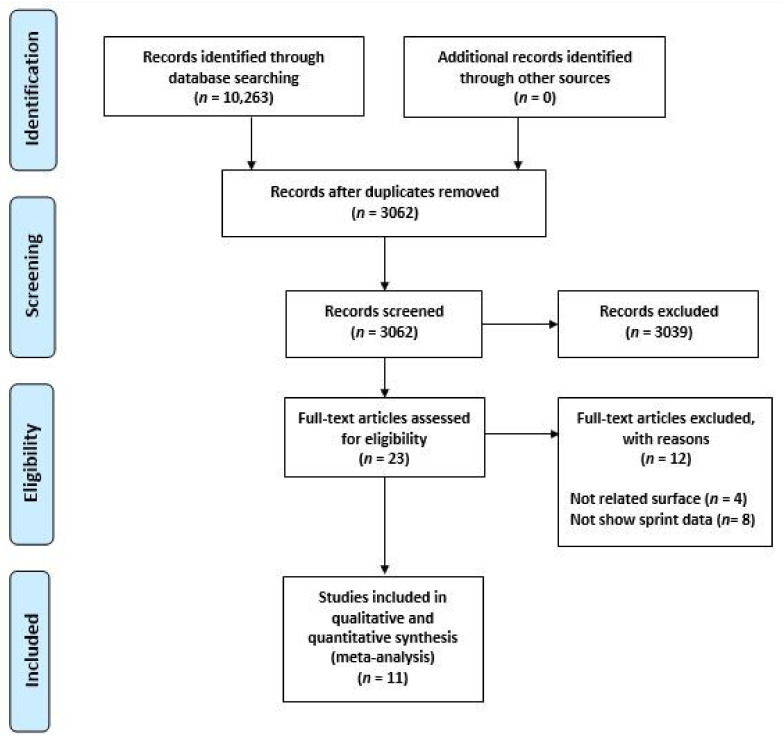
Flow diagram of the study.

The flow diagram in Figure 1 exhibits the process of study selection. From a total of 10,263 articles, 7201 were analyzed after removing 3062 duplicates, and 3039 publications were removed as they did not meet the eligibility criteria. Full-text papers (*n* = 23) were assessed for eligibility, with a further 12 of these being removed. Finally, 11 studies were included [11,12,18,21,25,26,27,28,29,30,31]. In total, the sprint speed of 252 players was evaluated on natural turf, artificial turf, or sand.

### 2.3. Data Extraction and Quality Assessment

The following variables were abstracted into a preformatted spreadsheet: authors, year of publication, characteristics of the study participants (number (*n*), age, % females, sport, training level), surface variables (natural turf, artificial turf, and sand), and method of sprint assessment (test type, measurement, distance, duration and type of the intervention). Furthermore, data extraction, quality assessment, and determination of the risk of bias were performed independently and in duplicate by two investigators (A.M.) and (J.S.), using PEDro Scale according to previous research [24]. Discrepancies were solved by discussion leading to consensus or through consultation with a third reviewer (L.G.) in accordance with the Cochrane Collaboration Guidelines [32]. 

### 2.4. Data Synthesis and Analysis

The meta-analysis and statistical analyses were performed using Review Manager software (RevMan 5.3; Cochrane Collaboration, Oxford, UK) and Comprehensive Meta-analysis software (Version 2; Biostat, Englewood, NJ, USA). 

To compare different surfaces, the size of the effect of the surfaces was also calculated by the difference in sprint speed (m/s) before any training or match on each surface, natural and artificial turf, or natural turf and sand. On the other hand, to compare the intervention effects on each surface, the differences in the mean (post- minus pre-intervention) values were analyzed. 

Each mean difference was weighted according to the inverse variance method [32]. Since sprint speed was assessed by different methods, the mean differences were standardized by dividing the values by their corresponding standard deviation. The standardized mean differences (SMD) in each trial were pooled with a random effects model [33]. In addition, the confidence interval (CI of 95%) was calculated to identify the magnitude of the changes and the effect size (ES; Cohen’s d). The ES was evaluated as follows: 0–0.2 = trivial, 0.2–0.5 = small, 0.5–0.8 = moderate, and 0.8 = significant [34].

**Figure 2:**
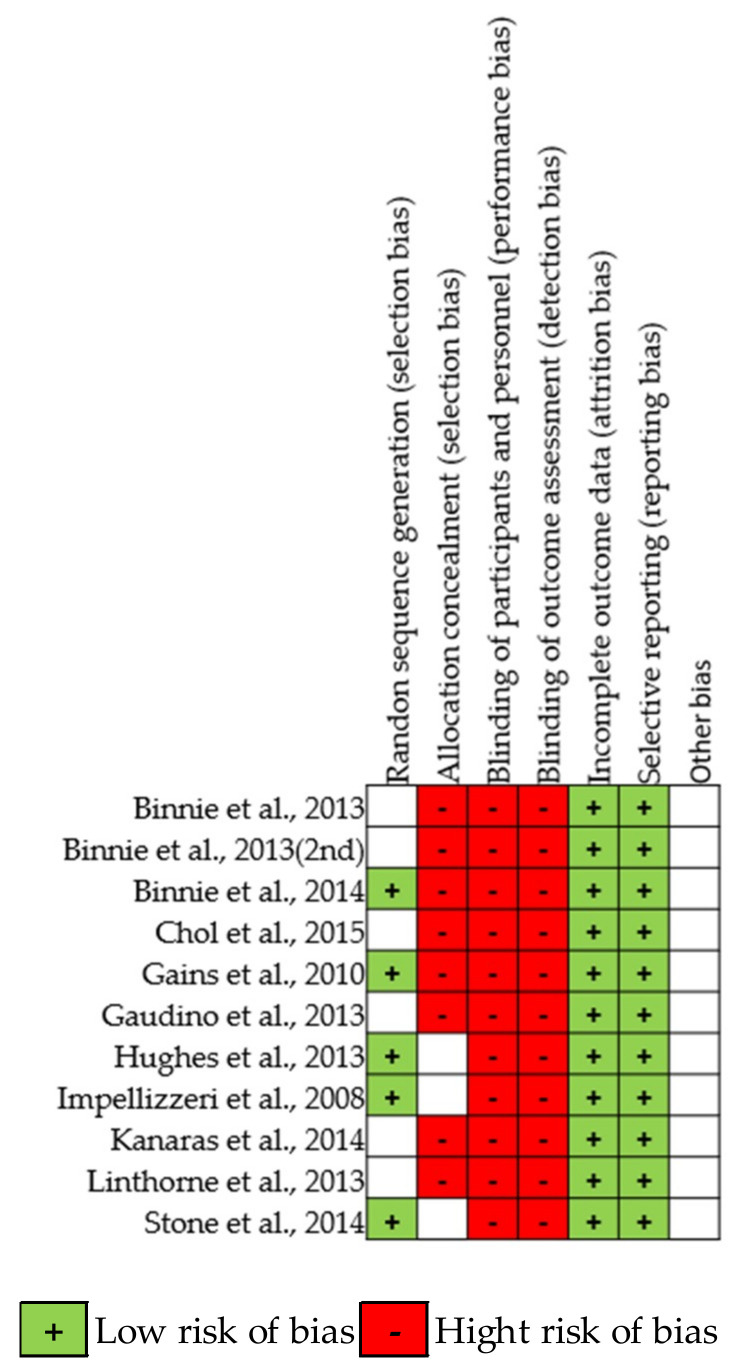
Risk of bias graph for each included study.

**Figure 3:**
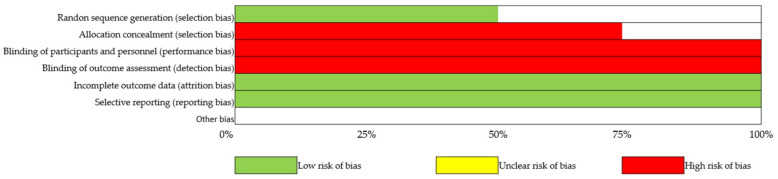
Risk of bias summary for all included studies.

Heterogeneity between studies was assessed using I^2^ statistics. The heterogeneity was considered low, moderate, or high if I^2^ = 25%, 50%, or 75%, respectively [35]. Publication bias was evaluated by a funnel plot asymmetry test and risk of bias summary and graphs (Figure 2 and Figure 3). A *p* value of less than 0.05 was considered statistically significant.

## 3. Results

**Figure 4:**
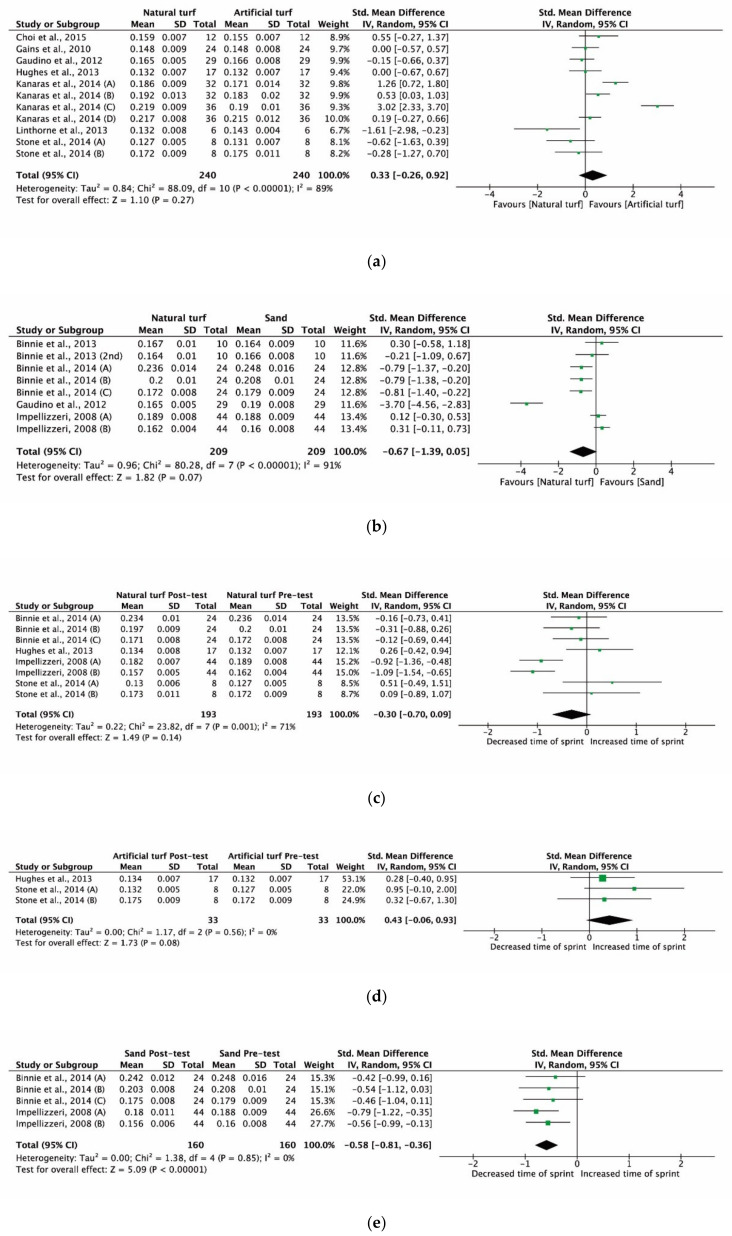
Standardized mean difference (SMD) between sprint times on: (**a**) natural vs. artificial turf; (**b**) natural turf vs. sand; (**c**) natural turf, pre- and post- sprint time assessment with different training methods; (**d**) artificial turf, pre- and post- sprint time assessment with different training methods; (**e**) sand turf, pre- and post- sprint time assessment with different training methods. Squares represent the SMD for each trial. Diamonds represent the pooled SMD across trials.

An evaluation of potential bias was made using a funnel plot for the SMD between post- and pre-test sprint speed on different surfaces; speed appeared symmetrical, suggesting the absence of a significant publication bias (Figure 4). Similar results were obtained for the evaluation of potential bias of the SMD in pre-test of sprints between natural turf and artificial turf or sand.

**Figure 5:**
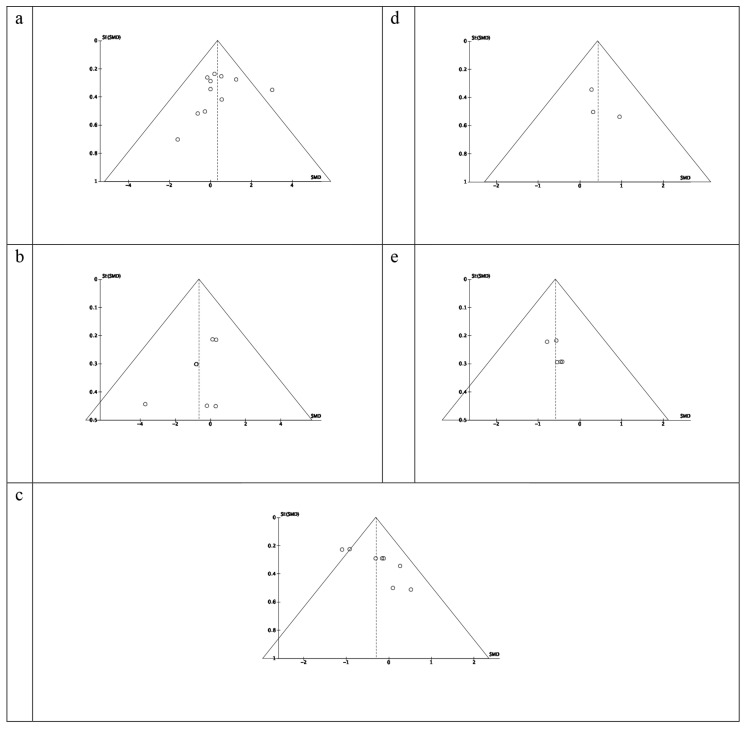
Funnel plots regarding: (**a**) natural vs. artificial turf; (**b**) natural turf vs. sand; (**c**) natural turf, pre- and post- sprint time assessment with different training methods; (**d**) artificial turf, pre- and post- sprint time assessment with different training methods; (**e**) sand turf, pre- and post- sprint time assessment with different training methods. The circles represent each of the studies. Squares represent the SMD for each trial. Diamonds represent the pooled SMD across trials.

The main characteristics and properties of the included studies are summarized in Table 1. The comparative analysis between natural and artificial turf (Figure 5a) revealed mixed results, with higher sprint speed obtained on artificial turf (ES = 1.30–3.05; *p* < 0.05) for football players [29] and higher sprint speed recorded on natural turf (ES = 1.83; *p* < 0.05) for rugby players [30]. On natural turf, the sprint speeds were better than on sand (Figure 5b) for netball and hockey players (ES = 0.80–0.82; *p* < 0.05; [25]) as well as for football players (ES = 3.85; *p* < 0.05; [11]), independent of the distance ran (Figure 4b). However, the global results did not show significant differences (z = 1.10, *p* = 0.27 for natural turf vs. artificial turf and z = 1.82, *p* = 0.07 for natural turf vs. sand).

Figure 5c shows the effects of the surface on sprint speed with different training methods. On natural turf, the included studies [12,25] revealed a reduction in sprint speed in both 10 m (ES = 0.93; *p* < 0.05) and 20 m (ES = 1.11; *p* < 0.05) sprints. The decrements on sprint speed after an 8-week pre-season conditioning program were higher on sand for all distances (*p* < 0.001) [25]. Finally, the studies that used a simulated soccer game protocol to test the effect of fatigue on natural and artificial turf [28,31] did not find a significant deterioration of sprint performance in relation to the sport surface (*p* > 0.05; Figure 4d). The global results did not show significant differences (z = 1.49, *p* = 0.14 for natural turf and z = 1.73, *p* = 0.08 for artificial turf). However, significant differences were found when considering different training methods on sand (z = 5.09, *p* < 0.001).

## 4. Discussion

In the present review, a meta-analysis was done with the aim of comparing sprint performance on natural turf, artificial turf, and sand. The selected studies showed significant differences in the maximum speed reached on artificial turf compared to natural turf and on natural turf compared to sand in the different subgroups analyzed. Thus, our analysis suggests that sprint performance on artificial turf is better than on natural turf, and on the latter, it is better than on sand. One of the reasons may be that the higher friction and rotational traction achieved on an artificial turf surface provide advantages to the performance of sprints and accelerations [36]. However, as many of the differences between sub-groups were not significant, the results are not conclusive, and further research is needed.

By analyzing the differences in sub-groups on natural and artificial turf, it was possible to find significant improvement on artificial turf only in studies examining more than 26 players. Similarly, these studies showed significant differences between results, in contrast with studies with smaller samples (PDifference). This indicates that large samples are necessary to draw inferences in this type of study. Likewise, practically, all the articles analyzed agreed that sprint performance on natural and artificial turf is very similar [11,21,28,30,31]. This could be due to the fact that the sprints examined were linear, whilst a performance difference depending on the surface appears with sprints including changes of direction, due to the different biomechanical responses on artificial turf [21,37,38].

Also, differences in sprint performance on different surfaces could depend on the presence of a ball during movement [27,29]. This suggests that for movement with a ball, the surface can influence sprint speed, inducing proper adjustments of the players [39]; in particular, hard surfaces allow reaching higher peaks of speed than soft surfaces [40,41]. The type of specific football shoe used on each surface can also affect performance, depending on how they influence players’ perception of the surface, by modifying the myoelectric activation of muscles [42]. However, these differences seem to be much smaller when technical actions, including speed performance, are analyzed in real games [39], as proven in this research. These results prove the homogeneity of natural turf and third-generation artificial turf football pitches, derived from the qualitative improvements that have been made in artificial surfaces [19]. Although no studies reported the mechanical properties of the surface, we can conclude that the differences between the percentages of force reduction on each surface were not high enough to generate an increase in speed derived from the reduction of the reaction forces following the partial absorption of the energy applied [16]. 

However, research on this topic requires further investigation. There is no consensus on the structural characteristics or quality parameters of the pitches considered in the research. In the case of natural turf, there are no established rules to analyze its mechanical properties in situ, as pointed out by Hughes et al. [28], and therefore, there is a lot of variability in these types of surfaces. Sleat et al. [43] highlighted the variability of the hardness of natural turf in amateur football pitches, finding significant differences in the movement patterns on different natural-turf pitches. In the case of artificial turf, Féderation Internationale de Football Association (FIFA) has established strict guidelines for artificial-turf football pitches to achieve specific functional characteristics and safeness. However, only the studies by Hughes et al. [28] and Stone et al. [31] indicated the accreditation of the pitches considered, which was FIFA 1 and 2 stars, respectively. Choi et al. [27] provided a data sheet of the product but did not make any reference to the mechanical properties of the surface.

Nonetheless, analyses done only considering if the surface of a pitch is in good state are not very informative, as they do not evaluate quantitative parameters. In this respect, Potthast et al. [44] suggested that the differences between different types of artificial turf surfaces could be larger than those between synthetic and natural surfaces. Sánchez-Sánchez et al. [10] proved the influence of structural support components and of the mechanical properties of artificial-turf football pitch surfaces on different test performance parameters and in simulated matches. Artificial-turf football pitches can vary in many parameters depending on the type of their sub-base and the type of fiber or infill, and each parameter can influence the performance of sport actions, affecting the traction on the pitch or the fatigue perceived [31]. In future researches, it will be necessary to identify the structural characteristics of football pitches and provide their mechanical properties through in situ pitch tests based on the FIFA or EN (European Norms) rules. 

Regarding the differences between natural turf and sand, the results of the present study prove better sprint speed on natural turf [11,25]. These differences confirm the hypothesis that softer surfaces require greater energy [45,46]. The deterioration in sprint performance on sand can be due to lower muscle–sinew efficiency [47] or to greater hip and knee flexion [48], as a consequence of a larger impact reduction on sand, even more pronounced during the acceleration phase or in short sprints, because of longer contact times [49]. 

The importance of this type of sport actions in sports like football, hockey, or rugby has been widely proven, with a high number of high-intensity actions performed on hard surfaces compared to sand, suggesting a higher dependency of creatine phosphate [14]. Instability on sand and cushioning caused by it seem to be responsible for a lower capacity to run at high speed on this surface [50]. On sand, greater impact absorption leads to a lower efficiency of the stretch–shortening cycle, with a reduced reuse of the stored elastic energy [51], which worsens the performance of sprint actions, as proven in the present meta-analysis.

However, the different effects of sand and natural turf surfaces could be associated with different adaptations of the neuromuscular system that they induce during training. Also, our meta-analysis revealed the influence of the type of training on the improvement of sprint performance, with plyometric training [12] inducing significant improvements on both sand and natural turf, in to relation to the improvement of the stretch and shortening cycle that it promotes. However, a greater effect on sprint speed improvement was identified on natural turf due to high stretch loads, which increase the pre-contraction activation state and activate the stretch reflex, thus favoring explosive concentric muscle contractions [52]. Despite this, the improvements highlighted on sand, associated with less stress on the musculoskeletal system, which limits muscle damage [12,51], indicate that this surface is adequate for rehabilitation or pre-season training. On the other hand, our meta-analysis did not find any differences in the fatigue test in football players on artificial and natural turf [28,31]. These results prove the similarity of these two surfaces, thanks to the amelioration of the quality of third-generation artificial-turf pitches [19] and to the increased familiarization of the players with this surface over the last years [53]. Figure 4a–d confirms that no overall effects were found. Only data from two papers showed a significant improvement in sprint speed after training on sand. The absence of studies on the influence of fatigue on sprint performance on sand prevented a comparison with results obtained on natural and artificial turf. However, studies that evaluated biomarkers showed higher intensity required by training on sand compared to natural turf, without affecting recovery 24 h after the test [26]. For this reason, future studies must analyze if this higher intensity on sand affects the immediate performance of sprint actions. 

## 5. Conclusions

In conclusion, the playing surface is a determining variable affecting the performance of sprint. Thanks to their qualitative improvement, artificial-turf pitches are similar to those in natural turf as regards their effect on sprint speed. The high impact absorption of sand represents the main factor deteriorating sprint performance. Despite this, sand surfaces do not prevent improvements. 

As for the limitations, of this study the high heterogeneity of the results observed when comparing different surfaces may be due to the limited time available to produce force during sprint on each surface. Other meta-analyses have shown high heterogeneity of results when different methods were compared [54].

Future research must include control variables to determine the effects of the structural characteristics of artificial- and natural-turf pitches, as well as their mechanical properties. However, the results of this meta-analysis do not show performance differences, in the case of sprint speed. In fact, the results show a better performance on artificial turf in some sub-groups. Previous studies showed that artificial turf also does not cause more injuries and can even reduce them [55]. Therefore, even though the effect of artificial turf on speed could in principle affect a game result, until now, scientific investigation suggests that it is an ideal surface to substitute natural turf in unfavorable economic situations or adverse climates, without negative repercussions on sprint performances 

## Figures and Tables

**Table 1:** Main characteristics of the studies included in the meta-analysis.

Study, Year of Publication		*n*	♀ (%)	Age (Years)	Sport	Level	Surface	Test	Intervention	Recorded Distance *
Binnie et al. (2013a)	-	10	33	21.15 ± 2.70	Netball and hockey	Well-trained	Natural turf and sand	RSA 8 × 20 m	-	20 m
Binnie et al. (2013b)	-	10	20	21.40 ± 1.80	Netball and hockey	Well-trained	Natural turf and sand	RSA 8 × 20 m	-	20 m
Binnie et al. (2014)	(A)	24	100	20.05 ± 5.70	Netball and hockey	Well-trained	Natural turf and sand	20 m sprint test	8-week pre-season conditioning program	5 m
	(B)									10 m
	(C)									20 m
Choi et al. (2015)	-	12	0	21.20 ± 2.00	Rugby	Semi-professional	Natural and artificial turf	40 m sprint test	-	40 m
Gains et al. (2010)	-	24	0	18.80 ± 0.40	American football	Elite (2nd division)	Natural and artificial turf	40 yd sprint	-	40 yd (36.6 m)
Gaudino et al. (2013)	-	29	0	19.00 ± 1.00	Soccer	Elite	Natural turf, artificial turf and sand	12 m sprint	-	12 m
Hughes et al. (2013)	-	17	0	22.80 ± 2.10	Soccer	Semi-professional	Natural and artificial turf	60 m sprint	soccer simulation protocol (SSP)	60 m
Impellizzeri et al. (2008)	(A)	44	0	25.00 ± 4.00	Soccer	Amateur	Natural turf and sand	10 m sprint test	4-week plyometric training	10 m
	(B)							20 m sprint test		20 m
Kanaras et al. (2014)	(A)	32	0	14.00 ± 0.50	Soccer	Amateur	Natural and artificial turf	multidirectional 30 m sprint test	-	30 m
	(B)					(Adolescent)				10 m
	(C)	36	0	12.00 ± 0.50	Soccer	Amateur	Natural and artificial turf	-		30 m
	(D)					(Children)				10 m
Linthorne et al. (2013)	-	6	0	20.00 ± 2.00	Rugby	Amateur	Natural and artificial turf	30 m sprint test	-	30 m
Stone et al. (2014)	(A)	8	0	20.30 ± 1.40	Soccer	Elite (1st division)	Natural and artificial turf	60 m sprint test	soccer simulation protocol (SSP)	60 m
	(B)									10 m

* Recorded distance: includes assessment of the total and partial distances of the test.
